# Effects of COVID-19 lockdowns on fine particulate matter concentrations

**DOI:** 10.1126/sciadv.abg7670

**Published:** 2021-06-23

**Authors:** Melanie S. Hammer, Aaron van Donkelaar, Randall V. Martin, Erin E. McDuffie, Alexei Lyapustin, Andrew M. Sayer, N. Christina Hsu, Robert C. Levy, Michael J. Garay, Olga V. Kalashnikova, Ralph A. Kahn

**Affiliations:** 1 Department of Energy, Environmental, and Chemical Engineering, Washington University in St. Louis, St. Louis, MO 63130, USA.; 2 Department of Physics and Atmospheric Science, Dalhousie University, Halifax, NS, Canada.; 3 Earth Sciences Division, NASA Goddard Space Flight Center, Greenbelt, MD 20771, USA.; 4 Goddard Earth Sciences Technology and Research, Universities Space Research Association, Greenbelt, MD 21046, USA.; 5 Jet Propulsion Laboratory, California Institute of Technology, Pasadena, CA 91109, USA.; 6 Department of Atmospheric and Oceanic Science, University of Maryland, College Park, MD 20742, USA.

## Abstract

Lockdowns during the COVID-19 pandemic provide an unprecedented opportunity to examine the effects of human activity on air quality. The effects on fine particulate matter (PM_2.5_) are of particular interest, as PM_2.5_ is the leading environmental risk factor for mortality globally. We map global PM_2.5_ concentrations for January to April 2020 with a focus on China, Europe, and North America using a combination of satellite data, simulation, and ground-based observations. We examine PM_2.5_ concentrations during lockdown periods in 2020 compared to the same periods in 2018 to 2019. We find changes in population-weighted mean PM_2.5_ concentrations during the lockdowns of −11 to −15 μg/m^3^ across China, +1 to −2 μg/m^3^ across Europe, and 0 to −2 μg/m^3^ across North America. We explain these changes through a combination of meteorology and emission reductions, mostly due to transportation. This work demonstrates regional differences in the sensitivity of PM_2.5_ to emission sources.

## Teaser

Satellite-derived PM_2.5_ concentrations are produced and examined to assess the effects of COVID-19 lockdowns on PM_2.5_.

## INTRODUCTION

The onset of the coronavirus disease 2019 (COVID-19) pandemic resulted in unprecedented restrictions beginning in early 2020 in the form of economic shutdowns and limitations on mobility, in many places for extended periods of time. These unique conditions offer a rare opportunity to examine the effects of changes in human activity on air quality. Satellite images released early in the pandemic showed marked decreases in tropospheric nitrogen dioxide (NO_2_), particularly over China. For example, a 48% reduction in NO_2_ columns was observed over China in the 20 days after the Lunar New year compared to the 20 days prior (*1*). Anthropogenic NO_2_ sources are mainly combustion processes, with power plant and transportation emissions being major sources (*2*–*4*). Of interest is how the COVID-19 restrictions affected ambient fine particulate matter (PM_2.5_) concentrations, which are ranked as the leading environmental risk factor for mortality as part of the Global Burden of Disease (*5*).

PM_2.5_ estimates are of particular relevance given the emerging evidence that exposure to PM_2.5_ exacerbates the severity of COVID-19 infection systems and may increase the risk of death in patients with COVID-19 (*6*, *7*). However, the accuracy of epidemiological studies of these associations depends on the availability and accuracy of ambient PM_2.5_ data. Analysis of satellite observations is needed to supplement ground-based air quality monitoring networks that have limited spatial coverage and density in populated regions to better understand associations with health outcomes to inform decision-making activities (*8*).

PM_2.5_ is formed from a variety of sources, both anthropogenic and natural. Anthropogenic sources of PM_2.5_ include emissions from power plants and industrial activity, residential combustion, transportation, and agriculture. Natural sources include processes such as mineral dust, emission of biogenic volatile organic compounds (VOCs) from vegetation, wildfire smoke, and sea spray. These emissions can be either primary or secondary sources of PM_2.5_. Primary sources directly emit particles to the atmosphere. Primary components of PM_2.5_ include black carbon, primary organic aerosols, desert dust, and sea salt. The concentrations of these primary particles are considered to be generally linearly related to their emissions. However, the relationship between secondary sources and PM_2.5_ concentrations is much more complicated and can be highly nonlinear (*9*). Secondary components of PM_2.5_ include secondary inorganic and organic aerosols. Secondary inorganic aerosols include the sulfate-nitrate-ammonium system of aerosols, which are formed through the gas-to-particle conversion of sulfur dioxide (SO_2_), nitrogen oxides (NO*_x_* = NO + NO_2_), and ammonia (NH_3_) gases. This system, in particular, can be highly nonlinear in terms of the relationship between emissions and aerosol formation (*10*, *11*). Secondary organic aerosols are formed through the partitioning of oxidized VOCs to particles in the atmosphere. This system can also be highly sensitive to nonlinearities in the relationship between emissions of gases such as NO*_x_* and organic aerosol formation (*12*–*14*). Therefore, a substantial reduction in NO*_x_* emissions may not necessarily result in a substantial reduction in total PM_2.5_ mass.

In line with a nonlinear chemical response, initial studies examining the effects of the COVID-19 lockdowns on PM_2.5_ concentrations using ground monitor data and chemical transport modeling have found somewhat unexpected results. Several studies showed that even with the strict restrictions put in place in China, there were several severe haze episodes, particularly over northern China. These episodes were found to be caused by a combination of stagnant meteorological conditions and increased secondary aerosol formation from power plant emissions coupled with an increased atmospheric oxidation capacity, despite substantial reductions in transportation emissions and manufacturing (*15*–*20*). Initial studies examining air pollution over the United States and Europe found that, although there were mostly substantial decreases in NO*_x_* concentrations, there were little to no decreases in PM_2.5_ concentrations (*21*–*28*). These initial insights into the PM_2.5_ system based on sparse monitoring motivate a more comprehensive assessment that includes satellite aerosol observations.

Recent advances in satellite remote sensing offer increasingly precise and accurate information (*29*–*32*). Using methods developed over the past decade (*33*–*36*), we map global PM_2.5_ concentrations inferred using aerosol optical depth (AOD) from several satellite instruments, which are combined and related to PM_2.5_ concentrations using relationships between surface PM_2.5_ and total column AOD simulated with a chemical transport model (GEOS-Chem). Geographically weighted regression (GWR) is applied to predict and account for the residual bias with regional ground monitor data over China, Europe, and North America, areas with considerable ground monitor density, producing regional monthly mean estimates for January to April 2018, 2019, and 2020 (Materials and Methods). We focus our analysis on these regions to compare PM_2.5_ concentrations during the lockdown months in 2020 to the same time period in the previous 2 years (2018 and 2019) to assess the effects of restrictions on PM_2.5_ levels. Using sensitivity simulations conducted with the GEOS-Chem model, we examine the impacts on PM_2.5_ concentrations of possible emission reductions during the pandemic, particularly in the transportation sector, and assess the possible influence of natural variability in meteorology.

## RESULTS

Our global satellite-derived PM_2.5_ estimates during the onset of the COVID-19 pandemic in January to April 2020 are described in Materials and Methods and are shown in fig. S1. Ambient concentrations vary by more than an order of magnitude, reflecting a complex mixture of anthropogenic and natural sources, modulated by photochemical processing, atmospheric transport, and depositional loss. Below, we examine changes versus prior years for China, Europe, and North America where strict lockdowns occurred. Multiple lines of evidence indicate that the months with the largest emission reductions were February for China (*37*–*40*) and April for Europe and North America (*37*, *41*–*43*). We treat these time periods as regional lockdown months. The months of January for China and January to February for Europe and North America are treated as nonlockdown control months.

**Fig. 1:**
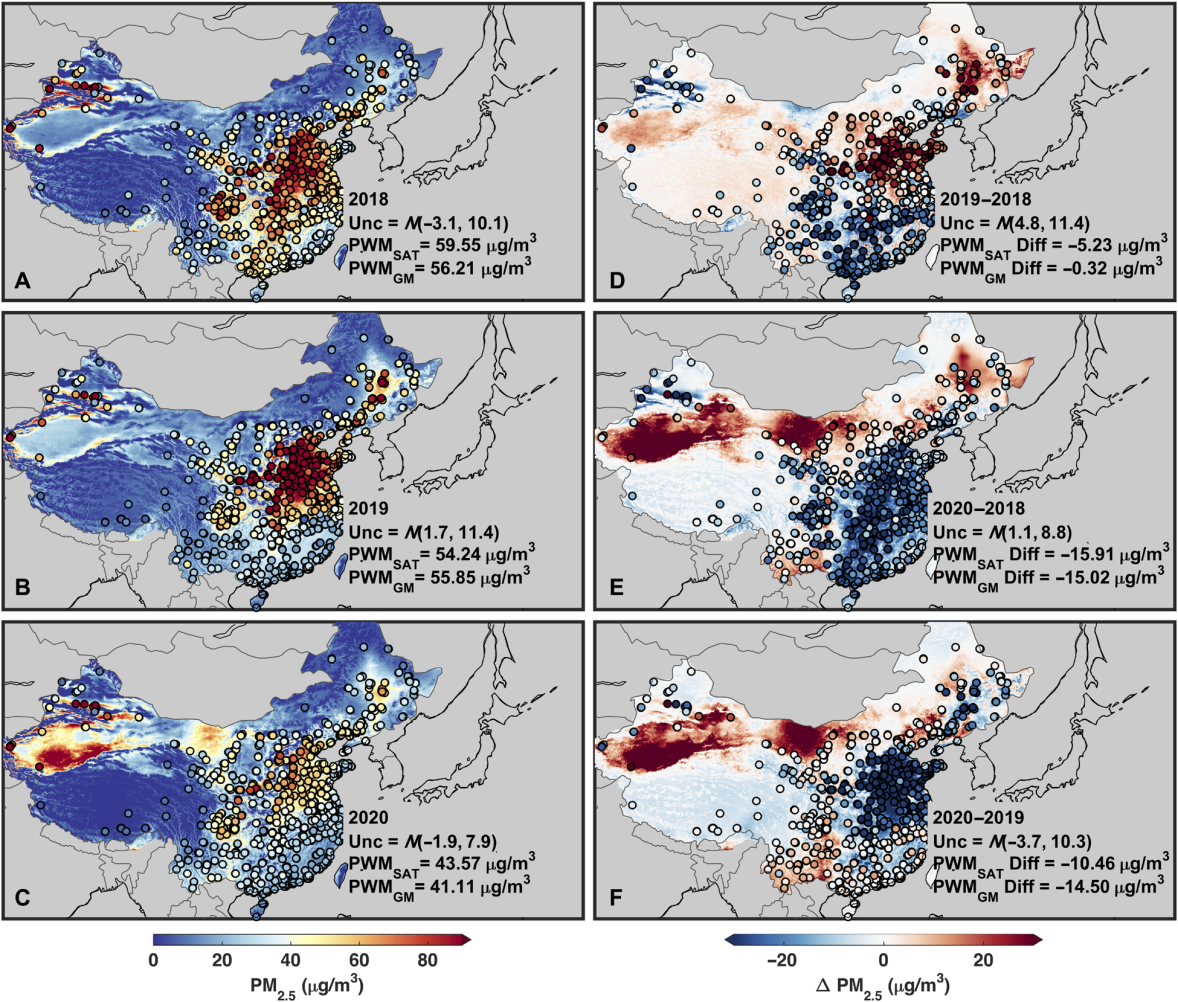
Monthly mean PM_2.5_ concentrations over China for February. The three panels to the left show the monthly mean satellite-derived PM_2.5_ concentrations over China for February 2018 (**A**), 2019 (**B**), and 2020 (**C**), with corresponding ground monitor PM_2.5_ concentrations overlaid in filled circles. Inset values show the normal distribution of uncertainty [*N*(bias, variance)] between collocated satellite and ground monitor PM_2.5_ concentrations, the population-weighted mean satellite-derived PM_2.5_ concentration collocated with ground monitor locations (PWM_SAT_), and the population-weighted mean ground monitor PM_2.5_ concentration (PWM_GM_). The three panels to the right show the 2019 to 2018 (**D**), 2020 to 2018 (**E**), and 2020 to 2019 (**F**) differences in February mean satellite-derived PM_2.5_ concentrations with the differences in ground monitor concentrations overlaid in filled circles. Inset values show the normal distribution of uncertainty [*N*(bias, variance)] between the collocated differences in satellite-derived PM_2.5_ concentrations and the differences in ground monitor PM_2.5_ concentrations, the population-weighted mean difference in satellite-derived PM_2.5_ values (PWM_SAT_ Diff) collocated to ground monitor locations, and the population-weighted mean difference in ground monitor PM_2.5_ concentrations (PWM_GM_ Diff).

The three left panels of Fig. 1 show the February mean satellite-derived PM_2.5_ concentrations for 2018 (Fig. 1A), 2019 (Fig. 1B), and 2020 (Fig. 1C) over China with available PM_2.5_ ground monitor concentrations overlaid in filled circles. The satellite-derived PM_2.5_ and ground monitor concentrations are overall consistent (population-weighted bias < 2 μg/m^3^, variance of 7.9 to 11.4 μg/m^3^). The spatial patterns between February 2018, 2019, and 2020 are similar, except for regions of northwest and north-central China exhibiting increases in PM_2.5_ concentrations in 2020 of approximately 25 μg/m^3^ and the North China Plain that exhibits a noticeable decrease in PM_2.5_ concentrations in 2020 (concentrations between 45 and 55 μg/m^3^) compared to the previous 2 years (concentrations between 65 and 80 μg/m^3^). The increases over northwest and north-central China are in close proximity to the Taklamakan and Gobi deserts that induce variability in desert dust as indicated by prior studies (*44*–*48*) and by our GEOS-Chem simulations discussed below. The decrease over the densely populated North China Plain is somewhat reflected in the overall population-weighted mean values with reductions of 11 to 15 μg/m^3^ in 2020 versus either 2018 or 2019. The difference plots (the three right panels of Fig. 1) reveal that this region shows increases of 10 to 15 μg/m^3^ between 2019 and 2018 (Fig. 1D) and then switches to substantial decreases of −25 to −20 μg/m^3^ for 2020 to 2018 (Fig. 1E) and −30 to −25 μg/m^3^ for 2020 to 2019 (Fig. 1F). The stronger decreases visible between 2020 and 2019 compared to 2020 and 2018 suggest some influence of natural variability. Examination of the nonlockdown month of January (fig. S2) reveals increases of +20 to +30 μg/m^3^ over parts of this region for the 2019 to 2018, 2020 to 2018, and 2020 to 2019 differences. These results suggest substantial decreases in PM_2.5_ concentrations in the North China Plain during the lockdown. Figure S3 shows a bar chart of population-weighted mean PM_2.5_ concentrations over the North China Plain for both the satellite-derived and the ground monitor concentrations for all 3 years during the lockdown period (February) and outside the lockdown period (January). The concentrations for January are higher for all 3 years (population-weighted mean between 80 and ~100 μg/m^3^) than during February with a small increase between 2019 and 2020. However, February shows a substantial decrease from ~70 μg/m^3^ in 2018 and 2019 to ~50 μg/m^3^ in 2020.

**Fig. 2:**
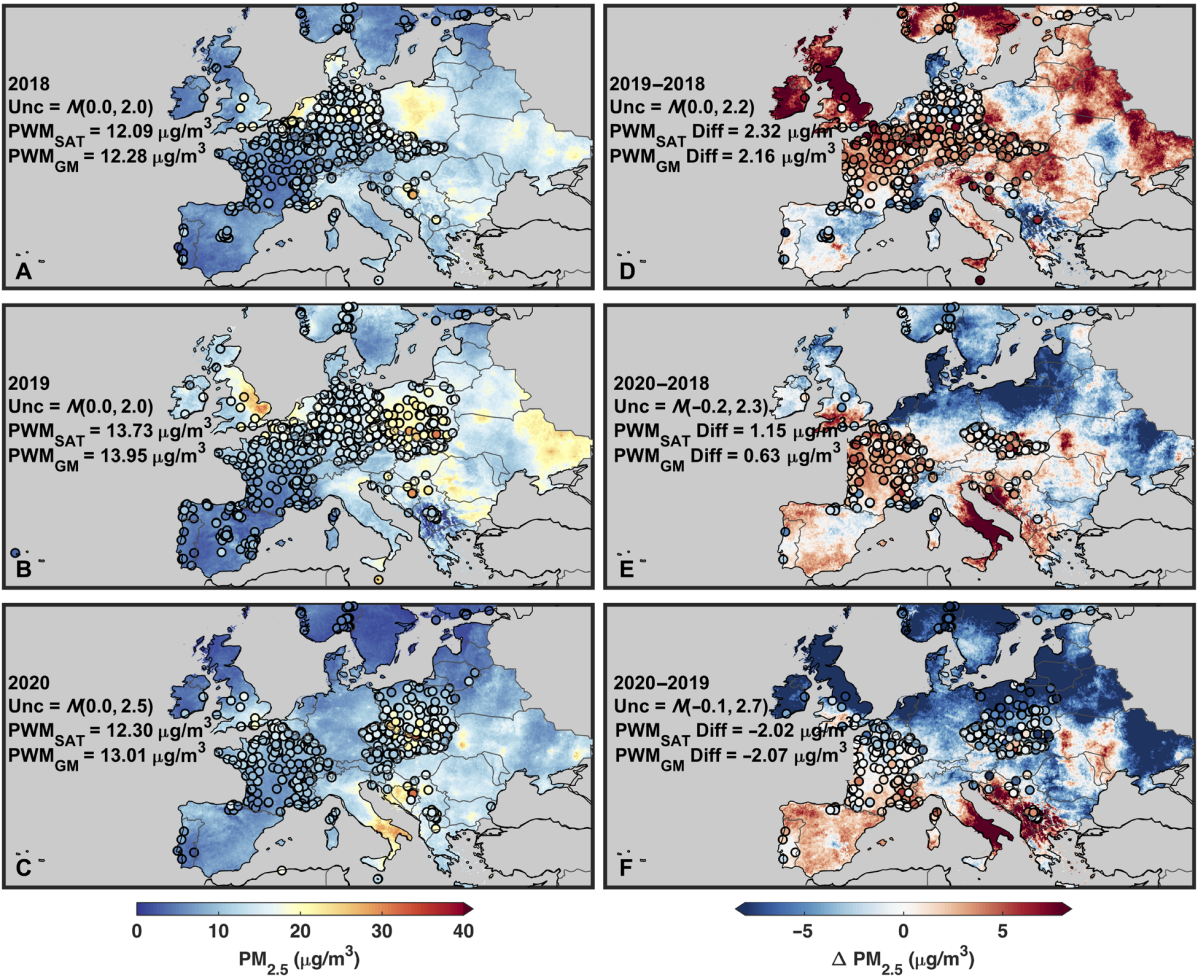
Monthly mean PM_2.5_ concentrations over Europe for April. Same as Fig. 1 but for April mean values over Europe. (**A**), (**B**), and (**C**) show the monthly mean satellite-derived PM_2.5_ concentrations for April 2018, 2019, and 2020, respectively, with corresponding ground monitor PM_2.5_ concentrations overlaid in filled circles. The three panels to the right show the 2019–2018 (**D**), 2020–2018 (**E**), and 2020–2019 (**F**) differences in April mean satellite-derived PM_2.5_ concentrations with the differences in ground monitor concentrations overlaid in filled circles.

For the lockdown month of April over Europe (Fig. 2), the satellite-derived concentrations and ground-based measurements are again consistent (population-weighted bias within 0.1 μg/m^3^), with both datasets exhibiting lower PM_2.5_ concentrations in western than eastern Europe. COVID-19–related changes are not immediately obvious when comparing the April 2020 values with the April 2018 and 2019 values, except for weak signals near Benelux. Interannual differences in PM_2.5_ concentrations exhibit both positive and negative anomalies that depend strongly on reference year. Examination of the nonlockdown months of January to February (fig. S4) reveals even larger interannual differences than during the lockdown months.

**Fig. 3:**
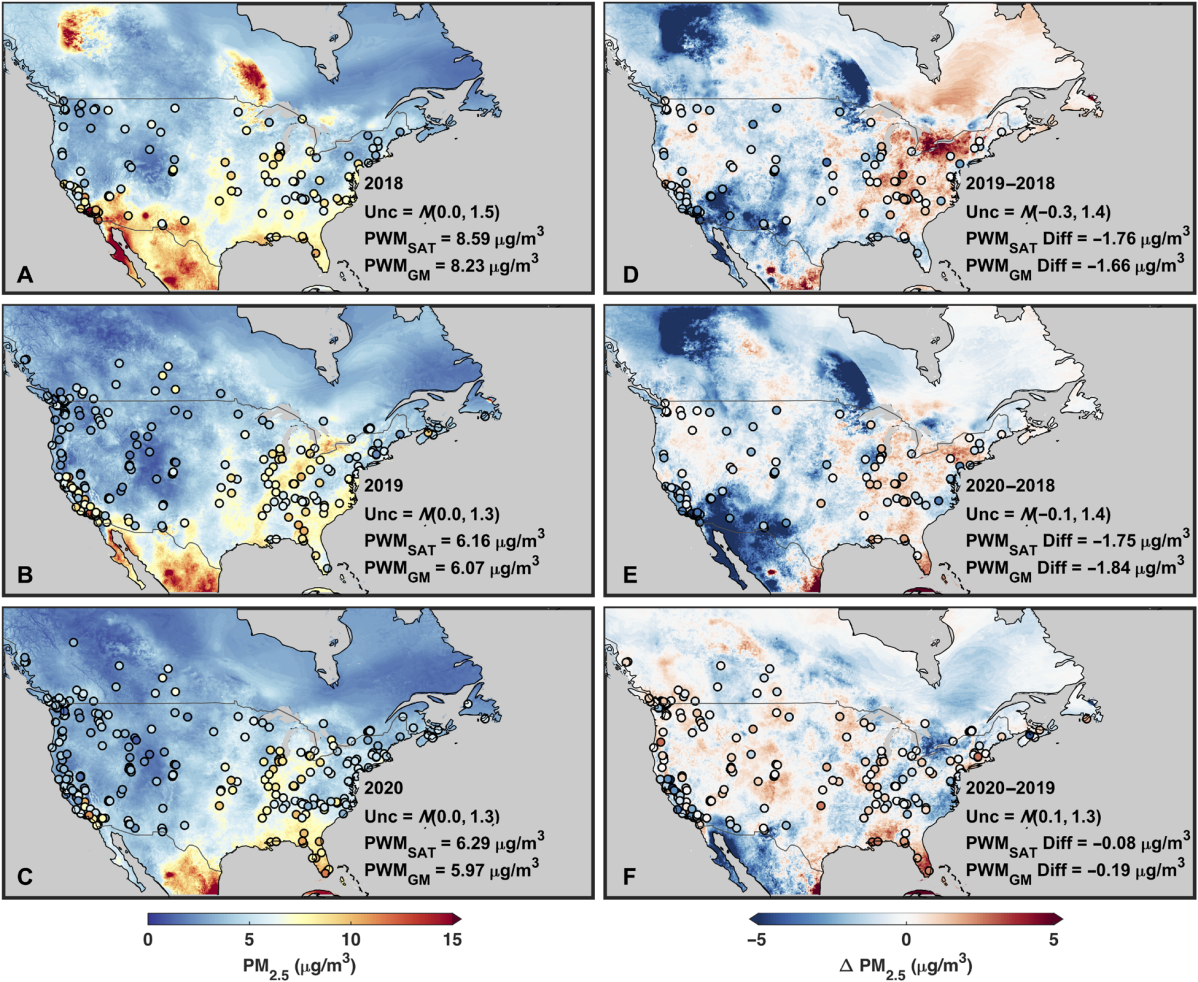
Monthly mean PM_2.5_ concentrations over North America for April. Same as Fig. 1 but for April mean values over North America. (**A**), (**B**), and (**C**) show the monthly mean satellite-derived PM_2.5_ concentrations for April 2018, 2019, and 2020, respectively, with corresponding ground monitor PM_2.5_ concentrations overlaid in filled circles. The three panels to the right show the 2019–2018 (**D**), 2020–2018 (**E**), and 2020–2019 (**F**) differences in April mean satellite-derived PM_2.5_ concentrations with the differences in ground monitor concentrations overlaid in filled circles.

For April over North America (Fig. 3), the satellite-derived and ground monitor concentrations are again consistent (population-weighted bias < 0.1 μg/m^3^). Interannual variability is again dependent on comparison years, with the smallest population-weighted mean differences between April 2020 and 2019, which are comparable to the bias in the estimates versus ground monitors. Overall, the interannual variability of PM_2.5_ concentrations and their population-weighted mean values during the lockdown are similar to the interannual variability and population-weighted mean values for the nonlockdown months (January to February 2020; fig. S5).

To investigate how changes in emissions during the lockdown periods contribute to the changes (or lack of) in PM_2.5_ concentrations, we conduct sensitivity simulations using the GEOS-Chem model with reduced transportation emissions and reduced NO*_x_* emissions across all anthropogenic sources (see Materials and Methods). The spatial pattern and extent of transportation reductions in 2020 are not well known, but reported reductions in fuel consumption (*37*, *43*, *49*) and observed NO_2_ columns (*41*, *42*, *50*) suggest regional transportation reductions ranging from 25 to 50% with larger local reductions in specific urban areas. Therefore, we conduct two test cases with commensurate emission reductions.

**Fig. 4:**
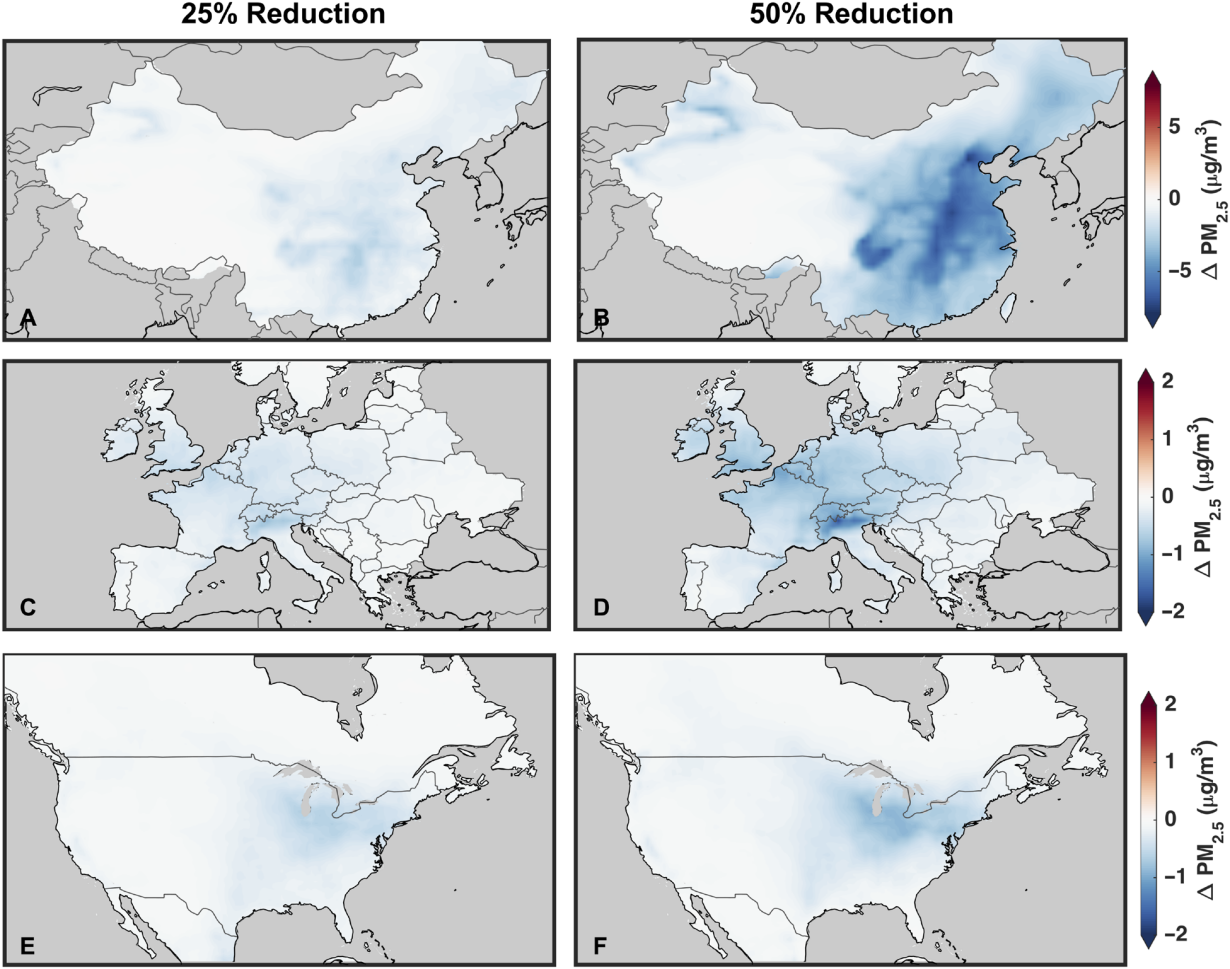
Change in simulated PM_2.5_ due to transportation emission reductions. Change in simulated surface PM_2.5_ concentrations due to a 25% (left column) and 50% (right column) reduction in transportation emissions of all species for February 2020 over China (**A** and **B**), April 2020 over Europe (**C** and **D**), and April 2020 over North America (**E** and **F**).

Figure 4 shows the change in simulated PM_2.5_ due to 25 and 50% reductions in transportation emissions, whereas fig. S6 shows the change in simulated PM_2.5_ due to 25 and 50% reductions in anthropogenic NO*_x_* emissions. Decreases in PM_2.5_ occur primarily near major urban and industrial regions in all scenarios, reflecting the distribution of transportation and industrial emissions modulated by atmospheric chemistry. Some simulated PM_2.5_ decreases such as over the North China Plain and central Europe align with observed changes (Figs. 1 to 3); however, emission reductions cannot explain most of the observed changes in either magnitude or distribution.

Examination of changes in simulated PM_2.5_ chemical composition offers insight into the magnitude of the effects. Reductions in all three regions are driven by reductions in nitrate aerosol due to reduced NO*_x_* emissions in all scenarios (figs. S7 and S8). These nitrate reductions are partially compensated by increases in sulfate and changes in ammonium, reflecting feedbacks on sulfur oxidation, aerosol thermodynamics, and heterogeneous chemistry. Similar feedbacks have been found in other sensitivity simulations to changes in NO*_x_* emissions (*15*, *51*). Feedbacks on the secondary organic aerosol system may also play a role due to changes in oxidants (*18*, *52*) and to aerosol acidity (*53*, *54*). The response found here highlights the nonlinearity between emission changes, the sulfate-nitrate-ammonium system, and PM_2.5_ formation processes (*55*, *56*).

**Fig. 5:**
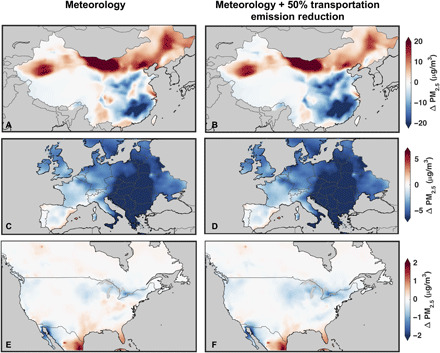
Change in simulated PM_2.5_ during the lockdown periods due to meteorology and emissions. The 2020 to 2019 difference in simulated surface PM_2.5_ concentrations due to meteorology (with emissions held constant; left column) and due to meteorology combined with a 50% reduction in transportation emissions (right column) for February over China (**A** and **B**), April over Europe (**C** and **D**), and April over North America (**E** and **F**).

Ambient PM_2.5_ concentrations are strongly influenced by meteorological conditions (*57*–*59*) that alter natural emissions, photochemical production, advection, and depositional loss. Therefore, we examine how natural variability due to meteorology coupled with reductions in emissions affects PM_2.5_. The left column of Fig. 5 shows results from a GEOS-Chem sensitivity simulation where the change in PM_2.5_ between 2020 and 2019 during the lockdown months is due solely to changes in meteorology, leaving emissions constant. The right column shows the change in PM_2.5_ between 2020 and 2019 during the lockdown months due to meteorology and a 50% reduction in transportation emissions. Meteorology has major influences as discussed in the Supplementary Materials (Supplementary Text). Over China, much of the spatial pattern in PM_2.5_ concentration differences seen in Fig. 1 can be explained by natural variability (Fig. 5A), except for a region showing decreases of <−20 μg/m^3^ toward southern China that is weaker in the satellite-derived estimates. Meteorology alone is insufficient to explain the reductions in the North China Plain; there is a mixture of increases and decreases up to ±10 μg/m^3^. When including the 50% transportation emission reductions (Fig. 5B), the small increases disappear, and decreases as low as −20 μg/m^3^ near the North China Plain develop, similar to the observed decreases in satellite-derived PM_2.5_ over the region. This suggests that the decreases observed over this region during the lockdown are driven by a combination of meteorology and emission reductions. In southern China, the overestimated reductions versus observations likely, in part, reflect within-country variation in the extent of emission reductions with the lockdown focus further to the north. Meteorological variability largely explains the observations of increases of approximately 25 μg/m^3^ in PM_2.5_ over northwest and north-central China, supporting that they are driven by natural variability in desert dust. Most of eastern and northern Europe shows decreases between −2 and −8 μg/m^3^ due to natural variability (Fig. 5C), which is comparable to the PM_2.5_ estimates in Fig. 2. One possible exception is the Benelux region that has weak changes due to meteorology but displays decreases in the PM_2.5_ estimates (Fig. 2). The combination of meteorology and reduced transportation emissions provides suggestive evidence of a regional COVID signal on top of larger natural variability. Over North America, meteorology appears to largely explain the small PM_2.5_ changes between 2019 and 2020, but there is some evidence that transportation emission reductions contribute to the strength of the observed reduction in the Great Lakes region (Fig. 3).

## DISCUSSION

The role of PM_2.5_ as the leading global environmental risk factor for mortality, coupled with emerging evidence of the association of ambient PM_2.5_ with COVID-19 outcomes, motivates attention to assessing ambient PM_2.5_ concentrations during the COVID-19 pandemic and understanding how societal interventions in response to the pandemic modulated ambient concentrations. Estimates of ambient concentrations during the pandemic are needed for epidemiological studies designed to better understand the relationship between COVID-19 and PM_2.5_ concentrations. However, gaps in ground-based monitoring, coupled with latency in availability of monitoring data, motivate alternative measures of PM_2.5_ from satellite remote sensing as inferred here. This activity builds upon a suite of advances across the satellite remote sensing community over the past decade with increasingly accurate and precise information at increasingly fine resolution. These satellite-derived PM_2.5_ concentrations offer timely information about air quality evolution during the COVID-19 pandemic.

Despite the widely reported pronounced reductions in ambient NO_2_ concentrations during COVID-19 lockdowns, we find that evidence of PM_2.5_ reductions specifically associated with COVID-19 lockdowns requires greater attention to discern, reflecting the complexity of PM_2.5_ sources and processes. The satellite-derived PM_2.5_ concentrations inferred here offer timely information in regions lacking public monitor data, whereas the addition of simulation and ground monitor data offers multiple constraints on the PM_2.5_ system. Comparison of PM_2.5_ during the lockdown months in 2020 to the same time period in the previous 2 years (2018 and 2019) reveals interannual variability that depends strongly on the reference year, implying a dominant meteorological influence in most regions. We find changes in population-weighted mean PM_2.5_ concentrations during COVID-19 lockdowns of −11 to −15 μg/m^3^ for China, −2 to +1 μg/m^3^ for Europe, and 0 to −2 μg/m^3^ for North America. Detection of changes in PM_2.5_ over North America and Europe may be complicated by the lower background levels in these regions, rendering the relative reductions difficult to discern. GEOS-Chem simulations provide supporting evidence of the combined influence of meteorology and emission reductions on features discernible during the COVID-19 lockdowns, particularly over the North China Plain with weaker signals over Benelux and the Great Lakes regions. The response of PM_2.5_ to COVID-19 lockdowns offers insight into the sensitivity of PM_2.5_ to transportation and other sources affected by the lockdowns. The strongest COVID-19 signal is a reduction of about 5 to 10 μg/m^3^ over the North China Plain during February, followed by reductions of a few micrograms per cubic meter during April near the Benelux region of northern Europe, followed by suggestive evidence of reductions of less than 1 μg/m^3^ in the Great Lakes region. Natural meteorological variability dominates elsewhere.

Long-term trends in emission reductions may also contribute to the PM_2.5_ reductions found here. Satellite-derived PM_2.5_ data indicate recent trends before COVID-19 of about −3.7 μg/m^3^ per year for China, −0.15 μg/m^3^ per year for Europe, and −0.28 μg/m^3^ per year for the eastern United States (*33*). These “business-as-usual” trends driven by emission controls are generally weaker than the observed anomalies during COVID-19 lockdowns but are nonetheless large enough to be a contributing factor.

This work demonstrates the complex relationship between secondary emission sources and PM_2.5_ concentrations; reductions in NO*_x_* emissions over all three regions are partially offset by nonlinearities in atmospheric chemistry. The weak sensitivity of PM_2.5_ to emissions from transportation is consistent with prior work. At the global scale, the transportation sector has been estimated to contribute 5 to 12% of population-weighted PM_2.5_ (*60*–*62*). At the regional scale, the contribution of the transportation sector to total PM_2.5_ has been estimated to be approximately 17% for China (*63*), approximately 14% for Europe (*64*), and less than 10% over the United States (*65*, *66*). Rather, the more dominant sources of PM_2.5_ from biofuel and mineral dust (*60*–*62*) were unlikely to have been strongly affected by the COVID-19 lockdowns. Future advances in quantifying emission changes associated with COVID-19 lockdowns will enable more precise estimates of the sensitivity of PM_2.5_ to its sources.

## MATERIALS AND METHODS

### Satellite AOD sources

Satellite AOD from six retrieval products from three satellite instruments is used to infer ambient PM_2.5_ concentrations. Twin Moderate Resolution Imaging Spectroradiometer (MODIS) instruments on the Terra and Aqua satellites provide daily global coverage. The Multiangle Imaging Spectroradiometer (MISR) instrument, which is also on the Terra satellite, provides global coverage about once per week.

The Dark Target (DT) algorithm (*29*) processes collection 6.1 MODIS radiances from both the Terra and Aqua satellites individually and is designed to retrieve AOD over dark surfaces (e.g., vegetated land surfaces and dark soils) by performing a simultaneous inversion of two visible (470 and 660 nm) and one shortwave-infrared (2120 nm) channel. The DT algorithm retrieves AOD at 550 nm at a spatial resolution of 10 km.

The Deep Blue (DB) algorithm (*30*) also processes collection 6.1 MODIS radiances from both Terra and Aqua individually and uses blue wavelength measurements where the surface reflectance over land tends to be much lower than at longer wavelengths, allowing for the retrieval of aerosol properties over both bright and dark surfaces. This is especially true over desert surfaces. The DB algorithm retrieves AOD at 550 nm at a spatial resolution of 10 km.

The Multiangle Implementation of Atmospheric Correction (MAIAC) algorithm (*31*) uses a dynamic minimum reflectance method to characterize spectral surface reflectance ratios by jointly processing MODIS radiances from the Terra and Aqua satellites. The MAIAC algorithm retrieves AOD at 466 nm over both bright and dark land surfaces including deserts. MAIAC reports AOD daily for MODIS Terra and Aqua observations at 466 and 550 nm at a fine spatial resolution of 1 km.

The MISR v23 algorithm (*32*) retrieves AOD from radiances measured by the MISR instrument, which observes Earth at nine different viewing angles and four spectral bands (446, 558, 672, and 866 nm), with a swath width of 380 km at all view angles that provides global coverage about once per week, every 9 days at the equator, and up to every 2 days near the poles. The MISR retrieval algorithm uses the same-scene multiangular views provided by the nine view angles to solve for surface and top-of-atmosphere reflectance contributions, providing AOD retrievals over bright and dark land surfaces without absolute surface reflectance assumptions. The MISR v23 algorithm retrieves AOD at 550 nm at a spatial resolution of 4.4 km.

### Ground monitor data

We use ground monitor data over North America and Europe from Open AQ (https://openaq.org/) for timely total PM_2.5_ mass information. To help promote data quality, we only use data from government monitoring sites. Over China, we use data collected by the Chinese government (http://beijingair.sinaapp.com/). Daily observations at each site are aggregated to monthly means, and sites with greater than 10 observations per month were included.

### GEOS-Chem simulations

We use the GEOS-Chem chemical transport model (*67*) v11-01 simulation described by Hammer *et al.* (*33*) as a data source for AOD and to represent the relationship of surface PM_2.5_ to total column AOD. The GEOS-Chem model solves for the evolution of atmospheric aerosols and gases using a detailed oxidant-aerosol chemical mechanism, emission inventories, and assimilated meteorological data. The assimilated meteorological data are from the Modern-Era Retrospective analysis for Research and Applications, Version 2 (MERRA-2) Reanalysis of the NASA Global Modeling and Assimilation Office (*68*). We conduct our simulation for January to April 2018 to 2020. We use the global spatial resolution of 2° × 2.5° and the nested spatial resolution of 0.5° × 0.625° over North America, Europe, and Asia with 47 vertical layers. The top of the lowest model layer is ~100 m. Regional anthropogenic emission inventories of aerosols and their precursors are used over the United States [EPA/NEI11 (*69*)], Europe (EMEP; www.emep.int), and China [MEIC (*70*)].

For our sensitivity simulations with reduced emissions from transportation and of NO*_x_*, we used the above simulation but with the Community Emissions Data System emission inventory, updated for the Global Burden of Disease–Major Air Pollution Sources project (CEDS_GBD-MAPS) (*2*). This global gridded inventory includes detailed sectoral information and enables consistent sectoral perturbations across all world regions. The transportation sector includes both road transportation and off/nonroad transportation emissions. Simulations with reduced NO*_x_* emissions include reductions in NO emissions from all anthropogenic sectors included in the CEDS inventory (agriculture, energy, industry, road transportation, off/nonroad transportation, residential/commercial, solvents, and waste). These sensitivity simulations build upon emerging information about emission changes during COVID-19 interventions. The transportation sector appears to have had some of the largest reductions (*49*, *71*), so we focus on that sector with reductions of 25 and 50%. The observed reductions in NO_2_ columns (*41*, *50*, *72*) indicate emission reductions primarily from transportation but potentially from the power and industry sectors as well. Thus, we conduct additional sensitivity simulations with total NO*_x_* reductions of 25 and 50% across all sectors. We conduct simulations with reductions across entire regions, given knowledge gaps in the spatial distribution of emission reductions.

### Estimating PM_2.5_ concentrations from satellite simulation and ground monitors

We first produce monthly geophysical-based estimates of surface PM_2.5_ concentrations using a combination of satellites and simulation following Hammer *et al.* (*33*). We then statistically fuse these geophysical-based estimates with regional ground monitor data following van Donkelaar *et al.* (*34*) to obtain monthly regional geophysical-statistical hybrid PM_2.5_ estimates for China, Europe, and North America.

To produce the geophysical-based PM_2.5_ estimates, we combine AOD from several satellite products (MODIS DB, MODIS DT, MISR, and MAIAC) based on their relative uncertainties with the global sun photometer network AERONET (V3) (*73*), which provides AOD measurements with high accuracy (uncertainty < 0.02). Simulated AOD from GEOS-Chem is also used as an additional AOD source; however, its contributions are mostly over northern regions where satellite retrievals are sparse (*33*). Satellite observations comprise 89% of the global combined population-weighted AOD data for the February to April 2018 to 2020 period (individual contributions to the global population-weighted mean: 22% MAIAC, 16% MODIS DB Terra and Aqua, 14% MODIS DT Terra and Aqua, and 7% MISR).

To estimate surface concentrations of PM_2.5_ from satellite AOD, we use the local, coincident ratio of simulated surface PM_2.5_ concentrations to simulated total column AOD. This ratio is a function of the factors that relate PM_2.5_ mass to satellite observations of AOD (e.g., aerosol size, aerosol composition, diurnal variation, relative humidity, and the vertical structure of aerosol extinction) (*74*).

The inability to retrieve satellite AOD in the presence of snow or cloud cover introduces sampling limitations when calculating PM_2.5_ from satellite AOD (*75*). We use GEOS-Chem to address these sampling limitations by scaling the satellite AOD by the ratio of simulated monthly mean AOD to simulated AOD coincident with satellite AOD. This approach builds upon the assimilated meteorology and mechanistic representation of processes such as photochemical feedbacks and deposition that are included in GEOS-Chem.

We then use GWR following van Donkelaar *et al.* (*34*) to predict the bias between the monthly geophysical-based PM_2.5_ estimates and the regional ground monitor observations. The GWR is conducted at a 1-km resolution to calibrate monthly parameter coefficients based on comparison with coincident ground monitor observations. The bias predicted by the GWR is used to adjust the geophysical-based PM_2.5_ mass estimate to produce the monthly geophysical-statistical hybrid PM_2.5_ estimates. Figure S9 shows the resulting hybrid PM_2.5_ estimates compared to ground monitor data for North America, Europe, and China for January to April 2018 to 2020.

### Population estimates

Population estimates are from the Gridded Population of the World (GPW v4) database (*76*). The 2018 and 2019 population estimates were obtained by linearly interpolating between 2015 and 2020. Population-weighted mean PM_2.5_ values are calculated as a weighted average weighted by the population estimates for the same year.

## SUPPLEMENTARY MATERIALS

Supplementary material for this article is available at http://advances.sciencemag.org/cgi/content/full/7/26/eabg7670/DC1
